# Synergistic Antitumor Activity of SH003 and Docetaxel via EGFR Signaling Inhibition in Non-Small Cell Lung Cancer

**DOI:** 10.3390/ijms22168405

**Published:** 2021-08-05

**Authors:** Mi-So Jeong, Kang-Wook Lee, Yu-Jeong Choi, Yun-Gyeong Kim, Hyun-Ha Hwang, Seo-Yeon Lee, Se-Eun Jung, Sun-Ah Park, Jin-Hee Lee, Yong-Joon Joo, Sung-Gook Cho, Seong-Gyu Ko

**Affiliations:** 1 Department of Science in Korean Medicine, Graduate School, Kyung Hee University, Seoul 02447, Korea; smilehnm7@gmail.com (M.-S.J.); ehowlqk11@naver.com (Y.-J.C.); yun__gyeong@naver.com (Y.-G.K.); se2435321@naver.com (H.-H.H.); dltjdus0225@naver.com (S.-Y.L.); dona9502@naver.com (S.-E.J.); jinhee2770@hotmail.com (J.-H.L.); kicku@naver.com (Y.-J.J.); 2 Institute of Safety and Effectiveness Evaluation for Korean Medicine, Kyung Hee University, Seoul 02447, Korea; kwleeband@gmail.com; 3 Department of Korean Medicine, College of Korean Medicine, Graduate School, Kyung Hee University, Seoul 02447, Korea; kingleggirl@hanmail.net; 4 Department of Biotechnology, Korea National University of Transportation, Chungbuk 27469, Korea; sunggook.cho@gmail.com; 5 Department of Preventive Medicine, College of Korean Medicine, Kyung Hee University, Seoul 02447, Korea

**Keywords:** non-small-cell lung cancer, epidermal growth factor receptor, docetaxel, SH003, anticancer

## Abstract

Epidermal growth factor receptor (EGFR) is overexpressed in lung cancer patients. Despite treatment with various EGFR tyrosine kinase inhibitors, recurrence and metastasis of lung cancer are inevitable. Docetaxel (DTX) is an effective conventional drug that is used to treat various cancers. Several researchers have studied the use of traditional herbal medicine in combination with docetaxel, to improve lung cancer treatment. SH003, a novel herbal mixture, exerts anticancer effects in different cancer cell types. Here, we aimed to investigate the apoptotic and anticancer effects of SH003 in combination with DTX, in human non-small-cell lung cancer (NSCLC). SH003, with DTX, induced apoptotic cell death, with increased expression of cleaved caspases and cleaved poly (ADP-ribose) polymerase in NSCLC cells. Moreover, SH003 and DTX induced the apoptosis of H460 cells via the suppression of the EGFR and signal transducer and activator of transcription 3 (STAT3) signaling pathways. In H460 tumor xenograft models, the administration of SH003 or docetaxel alone diminished tumor growth, and their combination effectively killed cancer cells, with increased expression of apoptotic markers and decreased expression of p-EGFR and p-STAT3. Collectively, the combination of SH003 and DTX may be a novel anticancer strategy to overcome the challenges that are associated with conventional lung cancer therapy.

## 1. Introduction

Lung cancer is the leading cause of cancer-related deaths worldwide, with approximately 1.8 million people being diagnosed with, and 0.6 million people dying of, lung cancer annually [1,2]. Depending on the stage and regional differences, the 5-year survival of patients with lung cancer ranges between 4% and 17% [3]. Based on their microscopic appearance, lung cancers are classified into non-small-cell lung cancer (NSCLC) and small-cell lung cancer (SCLC), which account for 85–90% and <20% of lung cancer cases, respectively [4]. For patients with NSCLC, chemotherapeutics, which are cisplatin-based cytotoxic drugs, and taxanes, such as paclitaxel and docetaxel (DTX), have been used as treatment [5,6].

DTX, a taxoid antineoplastic agent, facilitates the assembly of microtubules from tubulin dimers, and stabilizes microtubules by preventing depolymerization, leading to blockade of the cell cycle in the G2-M phase and induction of apoptosis [7,8]. As the induction of apoptosis is regarded as a promising strategy for cancer treatment, DTX has been applied in cancer chemotherapy, with an increased overall survival rate [9]. However, chemoresistance to DTX with epithelial–mesenchymal transition (EMT), tumor angiogenesis, or overexpression of ATP-binding cassette transporters (ABC drug transporters), remains the leading cause of treatment failure in patients with cancer [10,11]. Moreover, prolonged DTX therapy affects the healthy tissues and organs of patients with cancer, which causes severe adverse effects, including peripheral neuropathy, vomiting, anorexia, cachexia, and hair loss [12,13,14]. To overcome chemoresistance and adverse effects, several researchers have attempted to develop novel treatment strategies. Among them, combination chemotherapy has been proposed as a promising strategy to overcome cancer drug resistance [15,16]. Several clinical studies of DTX combined with herbal medicines have been conducted in order to determine their safety and efficacy in patients with cancer [17,18,19,20]. A few studies have demonstrated the survival benefit of chemotherapy combined with herbal medicines in patients with cancer [21,22]. Therefore, finding a novel partner for combination therapy with DTX may be a promising strategy for cancer treatment.

Epidermal growth factor receptor (EGFR) is a transmembrane receptor tyrosine kinase that is overexpressed in various malignant tumors, including NSCLC [23,24]. The binding of a ligand to growth factors, including EGF to EGFR, triggers downstream signaling pathways, which contribute to cell death suppression and promote cell proliferation [25]. Furthermore, activated EGFR via tyrosine phosphorylation at Y1068 initiates downstream signaling pathways, resulting in signal transducer and activator of transcription 3 (STAT3) activation that directly regulates cancer cell proliferation, metastasis, and angiogenesis [26,27]. The overexpression of EGFR, associated with poor prognosis, has been reported in patients with NSCLC [28]. Targeted therapy, using gefitinib or erlotinib as EGFR tyrosine kinase inhibitors (TKIs), is used in patients requiring first-line treatment, especially those with EGFR mutations [29]. Although a reasonable response to EGFR TKI treatment has been observed in patients with EGFR mutations, it has a negligible effect in patients with wild-type EGFR (EGFRwt) [30,31]. Therefore, the establishment of novel treatment strategies to improve the prognosis of patients with lung cancer with EGFRwt is essential. Recently, classical chemotherapeutic agents in combination with EGFR TKIs have been used to treat patients with NSCLC [32,33]. Furthermore, a clinical trial evaluating the efficacy and safety of gefitinib with DTX, as a second-line therapy for advanced or metastatic NSCLC patients with EGFRwt, is in progress (NCT01755923, https://clinicaltrials.gov, accessed on 10 January 2021). Several studies have suggested that combination therapy with DTX and anti-EGFR agents is likely to be effective against NSCLCs [34]. Thus, targeting EGFR activity with cytotoxic chemotherapy is a potential therapeutic method for EGFR wild-type lung cancer. 

The traditional herbal prescription, SH003, is composed of Astragalus membranaceus (Am), Angelica gigas (Ag), and Trichosanthes kirilowii Maximowicz (Tk). SH003 has been developed as a new drug, with anticancer properties against breast, prostate, and cervix cancers [35,36,37,38]. Previous studies have demonstrated that co-treatment with SH003 and anticancer drugs can overcome chemoresistance and improve drug sensitivity in cancer. SH003 not only enhanced the paclitaxel chemosensitivity of the paclitaxel-resistant cell line MCF-7, but also suppressed the growth of parental MCF7 breast cancer cells [39]. Therefore, we hypothesized that simultaneous treatment with SH003 and DTX could synergistically inhibit cancer growth. This study provides evidence of the mechanism involving EGFR in the anticancer effect of SH003 with DTX, and may herald new therapeutic methods using herbal medicine for lung cancer.

## 2. Results

### 2.1. Co-Treatment with SH003 and DTX Synergistically Inhibits the Growth of NSCLC Cells

**Figure 1:**
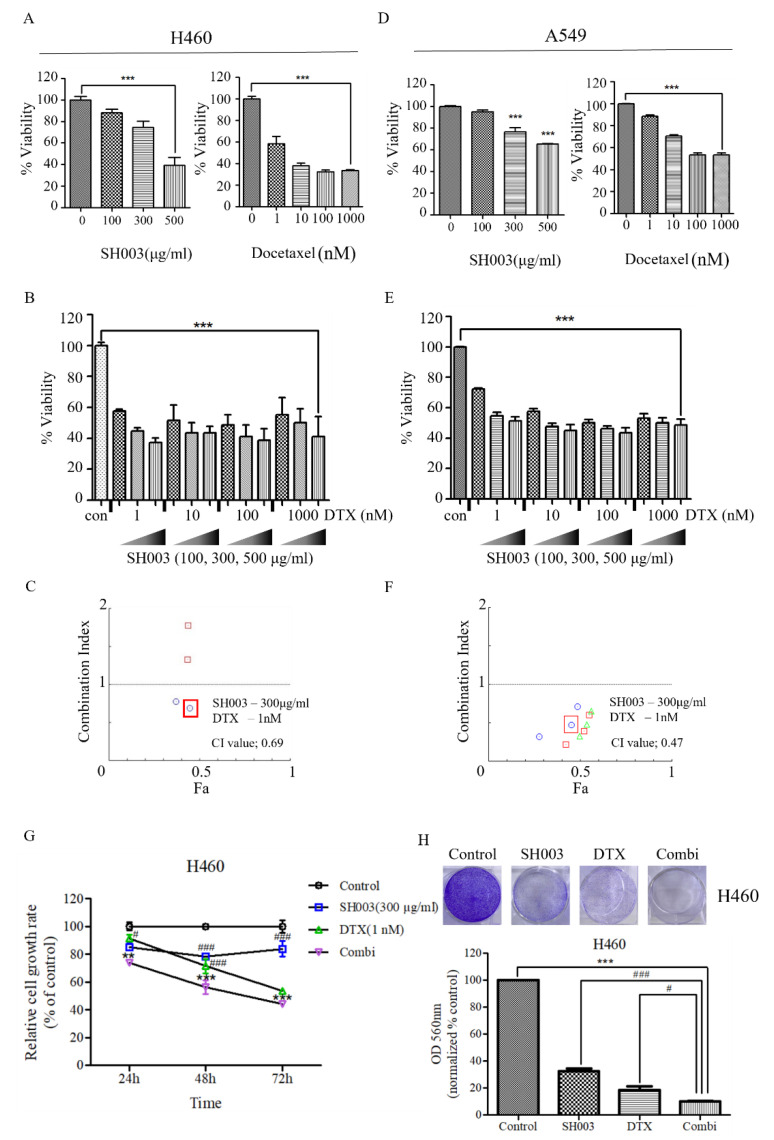
Effect of SH003 and docetaxel on the growth of H460 and A549 non-small-cell lung cancer cell lines. (**A**,**D**) Effect of single-drug treatment with SH003 or docetaxel on H460 and A549 cells. (**B**,**E**) Effect of combination treatment with SH003 and docetaxel on H460 and A549 cells. A cell viability assay kit was used to detect the proliferation of H460 and A549 cells treated with SH003 and docetaxel at various concentrations for 48 h. (**C**,**F**) Synergistic effect of SH003 and docetaxel was determined by combination index (CI) using CompuSyn software. CI < 1, CI = 1, and CI > 1 correspond to synergistic, additive, and antagonistic effects, respectively. (**G**) Proliferation of H460 cells treated with the SH003, docetaxel, or their combination, expressed as relative cell growth rate (% of control). Statistical significance was determined using the two-way ANOVA with Bonferroni post-tests. (**H**) H460 cells stained with crystal violet 5 days after treatment of SH003, docetaxel, or a combination of each drug. (**A**–**F**,**H**) Error bars are SEM of cells treated, *n* ≥ 3. Statistical significance was determined using the one-way ANOVA with Tukey’s test comparing control cells with SH003- or docetaxel-treated cells. ** *p* < 0.01, *** *p* < 0.001 versus control, and # *p* < 0.05, ### *p* < 0.001 versus combination.

First, we investigated the effects of SH003 and DTX on the viability of NSCLC H460 cells. As shown in Figure 1A, both SH003 and DTX inhibited cell growth in a dose-dependent manner. Moreover, the combination treatment of SH003 and DTX inhibited the growth of H460 cells more effectively than single-drug treatment (Figure 1B). To investigate whether the drug combination demonstrates a synergistic effect, the CI of the drug pair at several doses was calculated. Cell viability, determined using the WST assay, was analyzed using the CompuSyn algorithm (Figure 1C and Table 1). CI < 1 signifies synergism and CI > 1 signifies antagonism. Next, A549 cells were treated with various concentrations of SH003 and DTX. Similarly to H460 cells, the viability of A549 cells decreased in a dose-dependent manner (Figure 1D). The combination treatment of SH003 and DTX inhibited the growth of A549 cells more than treatment with one drug alone (Figure 1E). Based on the viability data of cells that were subjected to combination treatment, the synergistic effect was calculated using the CompuSyn algorithm (Figure 1F). As 300 µg/mL SH003 and 1 nM DTX showed the lowest CI (CI = 0.69) in H460 cells, these concentrations were used for further experiments. Furthermore, the proliferation of H460 cells that were subjected to the treatments for 24, 48 and 72 h was measured, to determine the relative growth rate (Figure 1G). The combination treatment of SH003 and docetaxel significantly inhibited the cell proliferation. In addition, the combination treatment significantly suppressed the growth of H460 cells in colony formation analyses (Figure 1H). These results indicate that SH003 and docetaxel effectively inhibit the viability of NSCLC cells.

### 2.2. Co-Treatment with SH003 and DTX Induces Apoptosis of NSCLC Cells

**Figure 2:**
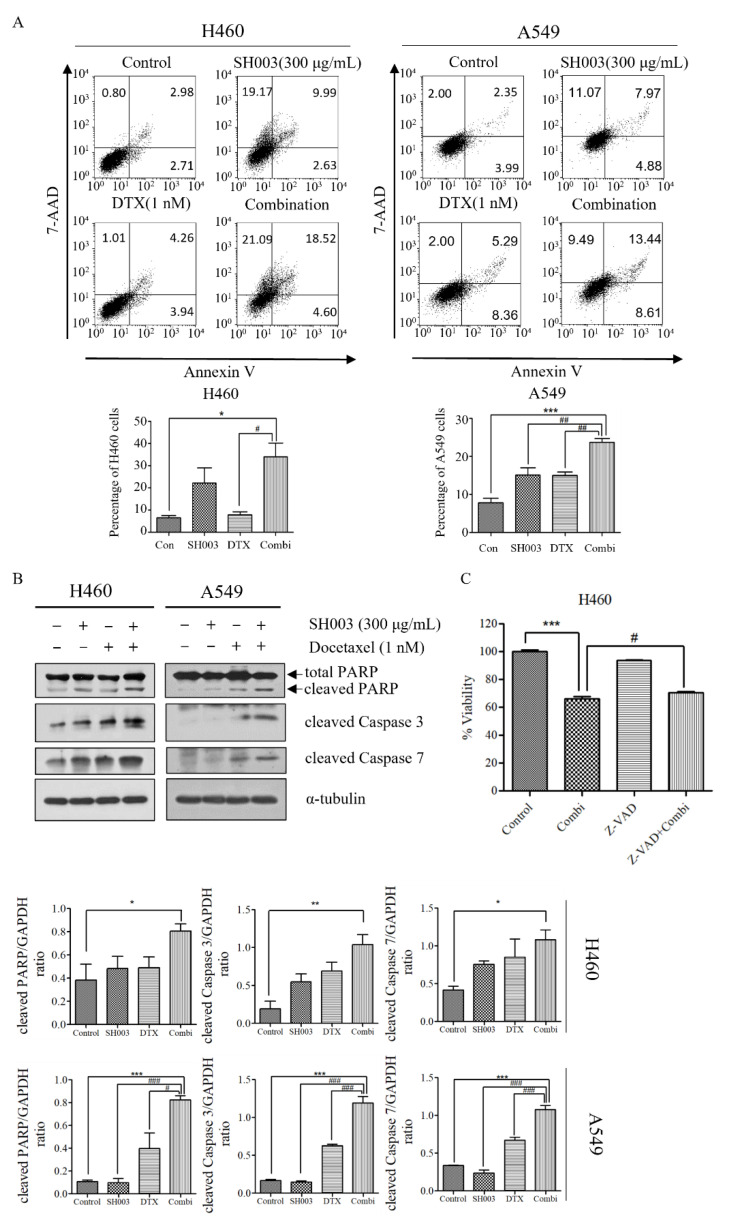
SH003 in combination with docetaxel induces apoptosis in H460 and A549 cells. (**A**) To detect apoptosis induced by SH003 and docetaxel, H460 and A549 cells were double stained with annexin V-FITC and 7-AAD for 24 h. (**B**) Expression levels of poly (ADP-ribose) polymerase (PARP), cleaved PARP, cleaved caspase-3, and cleaved caspase-7 were determined by Western blotting in H460 and A549 cells treated with SH003 and docetaxel for 24 h. Statistical significance was determined using the one-way ANOVA with Tukey’s test comparing the control; * *p* < 0.05, ** *p* < 0.01, *** *p* < 0.001, and comparing the combination; # *p* < 0.05, ## *p* < 0.01, ### *p* < 0.001. (**C**) H460 cells were pre-treated with 20 μM Z-VAD-FMK, a pan-caspase inhibitor, followed by their combination for 48 h. Cell viability was analyzed by WST-1 assay. Data are presented as the mean ± standard deviation (*n* = 3). Statistical significance was determined using the one-way ANOVA with Newman–Keuls multiple comparison test comparing control and combination treatment; * *p* < 0.05 and *** *p* < 0.001.

SH003 and DTX are known to exert anticancer effects by enhancing apoptosis [38,39]. To examine whether the combination treatment synergistically induced the apoptotic pathway, we measured apoptotic rates by double staining with annexin V and 7-AAD. The treatment with DTX for 24 h had no significant effect on the H460 and A549 cells compared with the control, whereas SH003 and combinatorial treatment induced cell death (* *p* < 0.05; Figure 2A). Under combinatorial treatment, apoptosis of H460 (34.07%) and A549 cells (23.74%) increased considerably. Consistently, the expression of apoptotic markers, including cleaved caspases 3 and 7, and cleaved PARP, increased synergistically in the H460 and A549 cells that were co-treated with SH003 and DTX (Figure 2B). Furthermore, Z-VAD-FMK, a pan-caspase inhibitor, was used to confirm the apoptotic effect of the combination treatment of SH003 and docetaxel. Z-VAD-FMK partially restored the viability of the H460 cells compared with the combination treatment condition (Figure 2C). These results indicate that cell death that was induced by combination treatment with SH003 and DTX involved the apoptotic pathway.

### 2.3. Combination Treatment with SH003 and DTX Induces Apoptosis and Suppresses Cell Proliferation by Inhibiting the EGFR/STAT3 Signaling Pathway

**Figure 3:**
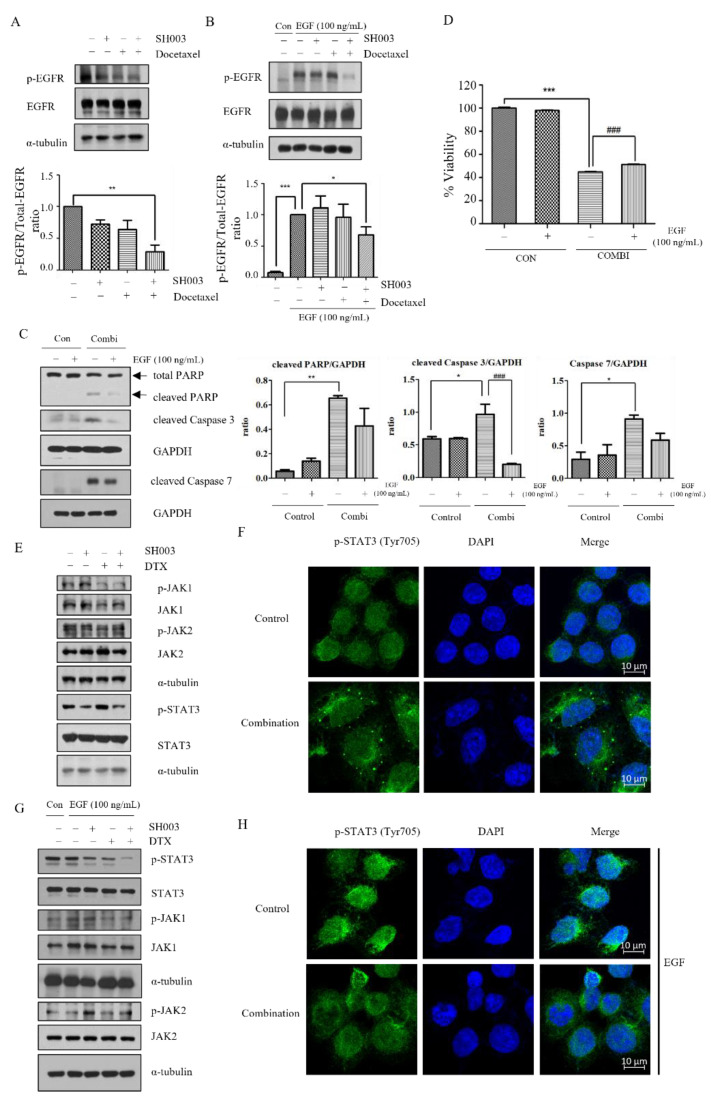
Combination treatment with SH003 and docetaxel downregulates the expression of p-EGFR and p-STAT3 in non-small-cell lung cancer. Lysates of H460 cells treated with 300 μg/mL SH003 or 1 nM docetaxel for 15 min were probed with the indicated antibodies. Further, α-Tubulin was used as the loading control. Quantification of proteins normalized to the control was performed, and the experiments were repeated three times. (**A**–**C**) Expression levels of proteins measured using Western blotting. (**B**–**D**) H460 cells pretreated with 100 ng/mL EGF, followed by treatment with 300 μg/mL SH003 and 1 nM docetaxel. (**D**) Viability of H460 cells after combined treatment with SH003 and DTX, and 100 ng/mL EGF for 48 h. (**E**,**G**) Expression levels of several proteins, including p-STAT3 (Y705) and p-JAK1/2, determined by Western blotting without or with 100 ng/mL EGF. (**F**) Intracellular distribution and expression level of STAT3 in H460 cells determined by immunocytochemistry without EGF. (**H**) H460 cells pre-treated with EGF (100 ng/mL), and subsequently treated with SH003 and docetaxel, at concentrations identical to those used in Western blotting. Combination treatment inhibited the translocation of STAT3 to the nucleus. The results shown here are representative of three independent experiments. Statistical significance was determined by one-way ANOVA with Tukey’s test comparing control. * *p* < 0.05, ** *p* < 0.01, *** *p* < 0.001 versus control and ### *p* < 0.001 versus the combination treatment with SH003 and DTX with 100 ng/mL EGF. EGF, epidermal growth factor; EGFR, epidermal growth factor receptor; STAT, signal transducer and activator of transcription.

Generally, EGFR is upregulated in NSCLC, and is involved in cell survival, proliferation, and drug resistance pathways. To examine whether SH003 and DTX induce apoptotic death of H460 cells, by targeting the EGFR pathway, we performed Western blotting. Our findings showed that the expression of p-EGFR (Y1068) decreased synergistically under combination treatment with SH003 and DTX (0.28; value normalized to that of the control) (Figure 3A). Furthermore, we investigated whether SH003 and DTX could downregulate EGFR phosphorylation that is activated by EGF in H460 cells. While EGF treatment increased the p-EGFR level, the combination treatment with SH003 and DTX significantly decreased it (Figure 3B). Western blotting revealed reduced levels of apoptosis proteins, including cleaved PARP and cleaved caspase-3, which had initially increased following co-treatment with SH003 and DTX, in H460 cells, after EGF treatment (Figure 3C). To determine the effect of EGF treatment on cell viability, we treated H460 cells with the combination of SH003 and DTX with or without EGF. The combination treatment reduced cell viability to 44%, but recovered it to 51% upon EGF treatment, showing a significant difference (Figure 3D). EGFR binding to several specific ligands activates multiple signaling pathways, resulting in cell proliferation. As one of the EGFR downstream pathways, here, we investigated the JAK/STAT3 signal transduction pathway. The expression of p-STAT3 (Y705) and p-JAK1, but not p-JAK2, was inhibited (Figure 3E). As the nuclear localization of p-STAT3 mediates the transcription of downstream genes for cancer proliferation, the localization of p-STAT3 in the cytosol was examined by IF staining in H460 cells. Compared with the control group, co-treatment with SH003 and DTX decreased the nuclear localization of p-STAT3 in H460 cells (Figure 3F). Furthermore, we investigated whether SH003 could downregulate EGFR phosphorylation that is activated by EGF. While EGF treatment increased the downstream proteins of p-EGFR, including p-JAK1 and p-STAT3, pretreatment with a combination of the drugs prevented this effect (Figure 3G). Further examination of the inhibitory effects of combination treatment revealed blockage of the nuclear translocation of p-STAT3 induced by EGF (Figure 3H). These findings suggest that combinatorial treatment with SH003 and DTX suppresses cell survival via the inhibition of the EGFR-induced JAK/STAT3 pathway.

### 2.4. Combination Treatment with SH003 and DTX Inhibited Tumor Growth in Xenograft Mouse Model

**Figure 4:**
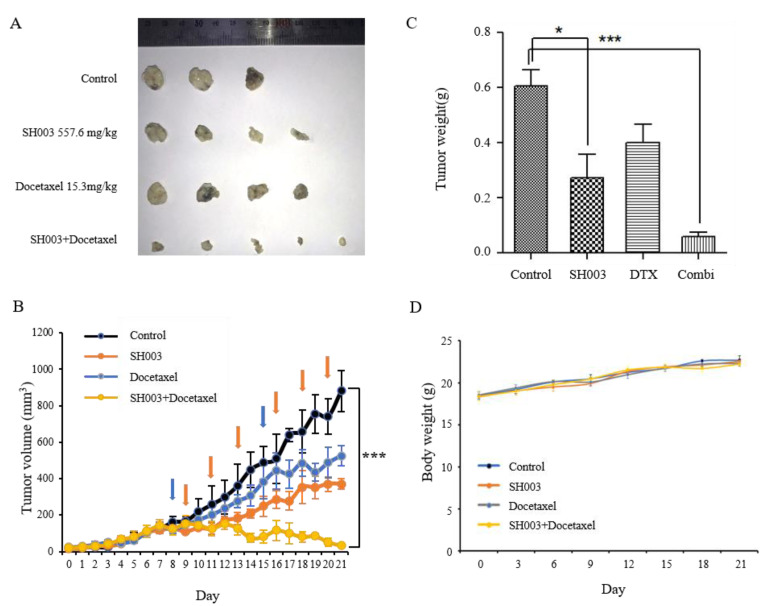
SH003 and docetaxel inhibited tumor growth in H460 xenograft mouse model in vivo. H460 cells (1 × 10^7^ cells/mice) were inoculated subcutaneously and the growth of the implanted tumor in different groups of mice was calculated longitudinally. (**A**) Measurement of xenograft tumors volume. (**B**) Orange and blue arrows indicate treatment with SH003 and docetaxel, respectively. At the beginning, the tumors in the four groups grew similarly in all aspects for 1 week, whereas tumor growth was retarded in the SH003- and DTX-treated group until 2 weeks. On day 21, the mean tumor volume in the combination group was smaller than that in the control xenograft group. Statistical significance was determined using the one-way ANOVA with Tukey’s test comparing control *** *p* < 0.001. (**C**) Tumor weights. Statistical significance was determined using the one-way ANOVA with Tukey’s test comparing control * *p* < 0.05 and *** *p* < 0.001 versus control. (**D**) The body weight was measured once every 3 days. The measured body weights between individual groups were similar.

**Figure 5:**
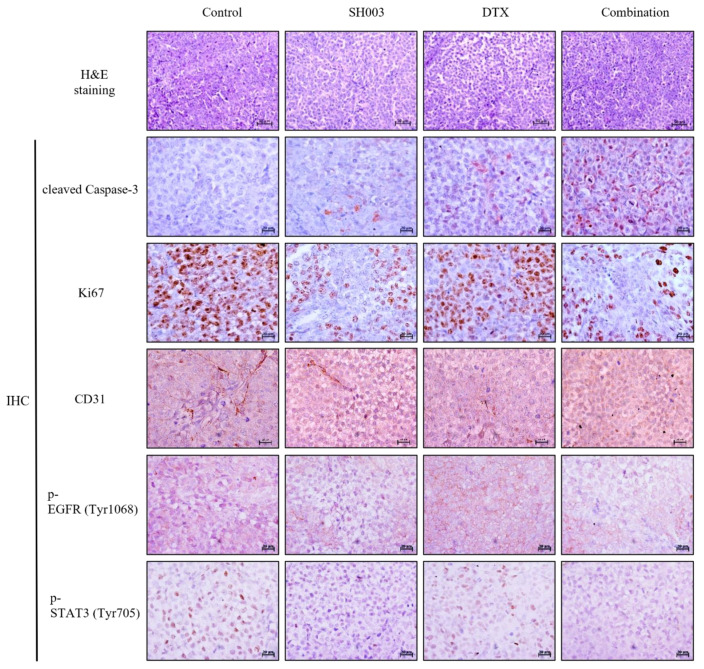
Immunohistochemical analysis of tumors treated with SH003 and docetaxel from H460 xenograft mouse model. Paraffin-embedded tumor sections were characterized by hematoxylin and eosin staining (scale bar, 50 µm) and immunohistochemistry for cleaved caspase-3, Ki-67, CD31, p-EGFR (Y1068) and p-STAT3 (Y705). Scale bar, 100 µm. These are representative images. STAT, signal transducer and activator of transcription.

Based on our results that SH003 and DTX synergistically inhibit cancer cell proliferation and promote apoptosis in vitro, we sought to determine the anticancer effect of SH003 and DTX in vivo. Tumor formation was induced by theinoculation of H460 cells into nude mice. The nude mice, bearing H460 xenografts, were treated with SH003 and DTX, alone or in combination (Table 2). Twenty-two days after H460 cell inoculation, the tumor-bearing mice were sacrificed, solid tumors were removed, and their weights were measured and analyzed. As shown in Figure 4A–C, SH003- or DTX-treated H460 xenograft tumors demonstrated a decrease in tumor volume (371 and 526.5 mm^3^, respectively) and weight (0.271 and 0.40 g, respectively) compared with the control group (880.5 mm^3^ and 0.604 g, respectively). The combination of SH003 and DTX exerted a considerably more significant effect in reducing the tumor volume (33.3 mm^3^; * *p* < 0.0001) and weight (0.059 g; * *p* < 0.05) compared with the single-agent treatment. Although SH003 and DTX demonstrated tumor suppression, there were no remarkable changes in the body weight of tumor-bearing mice in all the groups (Figure 4D). Thus, we confirmed that SH003 and DTX synergistically inhibited tumor growth, without causing toxicity in the mice in any of the groups. Tumor histological changes in xenograft mice were observed following H&E staining. As shown in Figure 5, tumor cells were densely populated in the tissues of the control group, but were sparse in the tissues of the SH003- and DTX-treated groups. The proliferation and apoptosis of tumor cells in NSCLC xenograft mice were assessed by Ki-67, CD31, and cleaved caspase-3 immunohistochemical staining. The expression of the representative markers of proliferation and angiogenesis, Ki-67 and CD31, was decreased compared with the control, by combinatorial treatment with SH003 and DTX. Furthermore, cleaved caspase-3, which is an important marker of apoptosis, significantly increased in co-treated tumor tissue compared with that in tumor tissue from the control group. Furthermore, the expression of p-EGFR (Y1068) and p-STAT3 (Y705) in co-treated tumors decreased compared with that in the tumors of the control group. Collectively, these findings suggest that SH003 and DTX inhibit tumor growth by promoting apoptosis and inhibiting the expression of p-EGFR (Y1068) and p-STAT3 (Y705).

## 3. Discussion

DTX is a conventional first-line chemotherapeutic agent for NSCLC; it exerts cytotoxic effects via the stabilization of microtubules and inhibition of mitotic cell division, which lead to cell death [40]. However, DTX is associated with a high recurrence rate and adverse effects, such as immune dysfunction [41]. One of the proposed approaches to overcome the limitations of conventional therapies is combinatory therapies with herbal medicine [16,42]. Studies have explored the efficacy of SH003 as an anticancer agent in several cancers, based on traditional medicine theories, without causing toxicity [43]. In the present study, we demonstrated that the combination of SH003 and DTX is an effective strategy for treating lung cancer.

Apoptosis is a promising target for treating several malignancies. Conventional chemotherapies abrogate tumor growth by activating caspase-dependent or -independent signaling pathways, resulting in DNA damage [39]. Newly developed anticancer drugs have been designed to target the apoptosis pathway [44]. The action mechanism of DTX-based therapies involve cell cycle arrest and apoptotic cell death [45]. However, acquired resistance to DTX causes cancer cells to evade apoptotic death, and this suggests that the promotion of apoptotic death of resistant cancer cells is a promising strategy in cancer treatment [46]. Considering that the herbal mixture SH003 exerts anticancer effects through apoptosis in several cancer cell types, and presents synergism with anticancer agents in inhibiting cancer cell growth, in the present study, we investigated the anticancer effects of the combination of SH003 and DTX against lung cancer [37,38]. Our data showed that SH003 improves DTX-mediated cell death in H460 cells, by increasing apoptosis with an increase in apoptotic marker proteins including cleaved caspase-7 and cleaved PARP. In addition to its apoptotic effect, the present study showed the inhibitory effect of SH003 on angiogenesis, by downregulating CD31 expression, which is a representative marker of angiogenesis. Previously, SH003 repressed tumor angiogenesis by blocking the binding of VEGF to VEGFR2 [47]. DTX also showed an inhibitory effect on VEGF expression. There is a need for further research to determine whether the combination treatment affects tumor angiogenesis. The finding of the present study suggests that co-treatment with SH003 and DTX has the potential for anti-angiogenesis activity. 

A reason for DTX resistance of cancer cells is EMT. EMT has key roles in the metastasis of cancer cells and their resistance to anticancer agents. In a previous study, the DTX-resistant NSCLC cell line A549/DTX showed EMT [48]. The expression of EMT markers, including MDR1, Bcl-2 and Bax, was increased in A549/DTX cells, highlighting the importance for novel strategies through increased drug sensitivity. Furthermore, the expression of the master EMT-inducing transcriptional factor ZEB1 was significantly increased in DTX-resistant human lung adenocarcinoma cell line (SPC-A1/DTX) [49]. SH003 reportedly enhances the effect of paclitaxel in MCF-7/PTX breast cancer cells, via the inhibition of MDR1 activity [38]. MDR is associated with chemotherapy-induced EMT and drug resistance. Considering the promising effect of SH003 with increased chemosensitivity through MDR1 inhibition, SH003 and DTX should be further researched in terms of EMT. 

EGFR belongs to the ErbB receptor tyrosine kinase family and forms homodimers or heterodimers after phosphorylation, and subsequently initiates an extensive intracellular signaling cascade [50]. Several lung cancer patients with poor prognosis exhibit EGFR overexpression and hyperactivation due to EGFR mutations. Therefore, EGFR has been suggested to be an important therapeutic target [51]. In the present study, combinatorial treatment with SH003 and DTX synergistically inhibited EGFR phosphorylation at Y1068. Furthermore, it decreased the p-EGFR level in response to EGF stimulation. The expression of apoptosis-related proteins increased during combination treatment with SH003 and DTX, and reduced after EGF treatment, leading to the recovery of H460 cell viability. This suggests that the combination treatment with SH003 and DTX could be a supplementary adjuvant treatment to downregulate EGFR activation.

As a crucial EGFR downstream effector, STAT3 can be activated by phosphorylation at the site of Y705, by the EGFR-induced signaling cascade, thereby leading to the transcriptional regulation of cell proliferation, angiogenesis, and metastasis [52]. With a reduction in the phosphorylation of EGFR, the phosphorylation of JAK1/STAT3 was also decreased in this study. STAT3 phosphorylation is regulated by JAK1/JAK2; however, we found that p-JAK1, but not p-JAK2, decreased in response to combinatorial treatment with SH003 and DTX. In another study, JAK2-independent STAT3 activation was observed when a p-JAK2 inhibitor was used [53]. Thus, the downregulation of STAT3 phosphorylation by SH003 and DTX is believed to occur via the JAK2-independent pathway. Similarly to the in vitro findings, our in vivo results indicated that the combination of SH003 and DTX suppressed tumor growth and decreased p-EGFR (Y1068) and p-STAT3 (Y705) expression compared with the control in the NSCLC xenograft model. Collectively, our findings demonstrate that the combination of SH003 and DTX is a potential treatment strategy to inhibit the growth of lung cancer, by targeting the EGFR and STAT3 pathways.

Small-molecule EGFR inhibitors, known as TKIs, have been used to treat patients with lung cancer. It has been shown that EGFR TKI monotherapy can benefit patients with NSCLC harboring EGFR mutations [54]. However, clinical trials have demonstrated low effectiveness of EGFR TKIs in patients harboring EGFRwt. In patients with EGFRwt NSCLC, the efficacy of gefitinib and erlotinib is limited, as evidenced by shorter progression-free survival (PFS) and a lower objective response rate than those achieved with cytotoxic chemotherapy [55]. Furthermore, the inferior effect of EGFR TKIs, in terms of PFS, has been found not only in first-line treatment, but also in second-line/third-line treatment [56,57]. However, it has been found that the addition of EGFR TKIs to chemotherapy has a survival benefit over chemotherapy alone in patients with EGFRwt [57]. In advanced NSCLC patients with EGFRwt, DTX is used as a second-line chemotherapy. As afatinib and DTX display considerable efficacy in treating advanced non-squamous NSCLC with EGFRwt, EGFR-targeted therapy with DTX is considered to have potential for NSCLC treatment [36]. In this study, the combination treatment with SH003 and DTX exhibited EGFR targeting. The first phase 1 clinical trial verified the safety and maximum tolerated dose of SH003 in patients with solid cancers (NCT03081819, https://clinicaltrials.gov, accessed on 1 March 2020). The present study showed that the SH003-mediated anticancer effect improves DTX-induced inhibition of lung cancer growth by targeting EGFRwt. Therefore, combination treatment with SH003 and DTX may have significant therapeutic potential for NSCLC patients with EGFRwt. Further, in vivo studies are necessary to elucidate whether SH003 can manage DTX-induced adverse effects, including peripheral neuropathy, anorexia, and cachexia, which commonly occur in chemotherapy recipients. 

**Figure 6:**
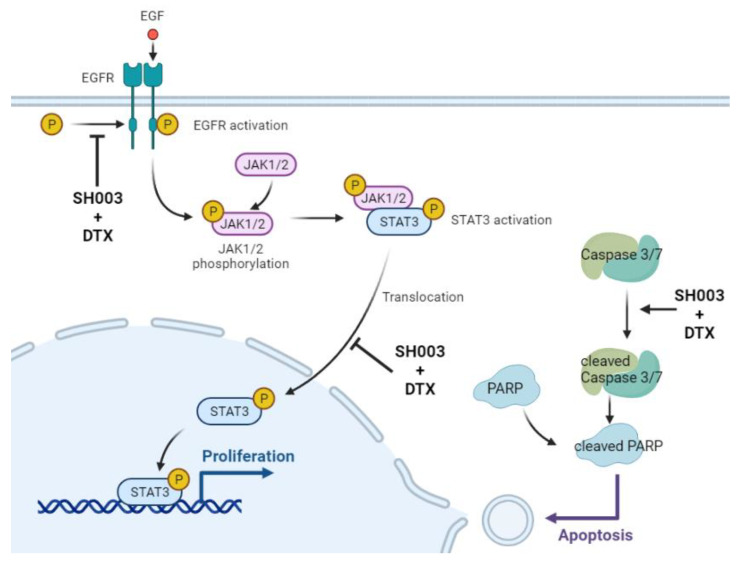
Overview of the proposed anticancer mechanism of SH003 in combination with DTX in H460 cells.

In this study, we elucidated the anticancer effect of SH003 alone or in combination with DTX against lung cancer. Although the anticancer effect of SH003 has been demonstrated previously, it is unclear if the compounds in SH003, in their active form, have an anticancer effect, as the components of other herbal compounds [58]. For example, some active compounds in SH003, such as formononetin and decursin, are known to have an anticancer effect [59,60]. However, given that SH003 contains diverse herbal components, it is difficult to determine whether the efficacy of SH003 is due to their combinatorial activity or one specific molecule. Thus, defining the action mechanism of these molecules is important. In our study, the compounds in SH003 and DTX in combination showed anticancer potential. Therefore, the combination of SH003 and DTX exerted a synergistic effect in the H460 cell line, and the results highlight the combination of SH003 and DTX as a promising complex herbal medication (Figure 6). However, further studies are required to discover and define the specific compounds that are involved in the anticancer effect.

## 4. Materials and Methods

### 4.1. SH003 and Chemicals

SH003, composed of Am, Ag, and Tk in a ratio of 1:1:1 was prepared as described previously [38]. Whole extracts were provided by Hanpoong Pharm and Foods Company (Jeonju, Korea) and manufactured following the guidelines of the Good Manufacturing Products (GMP). Dried extracts were dissolved in 30% ethanol and stored at −20 °C. DTX (Sigma-Aldrich, St. Louis, MO, USA) was dissolved in dimethyl sulfoxide (DMSO) and stored at −20 °C. EGF (Sigma-Aldrich) and Z-VAD-FMK (R&D Systems, Inc., Minneapolis, MN, USA) were dissolved in distilled water.

### 4.2. Cell Lines and Culture

The human NSCLC cell lines H460 (no. 30177, Korean Cell Line Bank, Seoul, Korea) and A549 (no. 10185, Korean Cell Line Bank, Seoul, Korea) were grown in RPMI 1640 medium (WelGENE, Daegu, Korea) supplemented with 10% fetal bovine serum (FBS; JR Scientific, Inc., Woodland, CA, USA) and 1% penicillin/streptomycin solution (WelGENE). Cells were incubated at 37 °C in a humidified atmosphere with 5% CO_2_.

### 4.3. Cell Viability Assay

Cell viability was measured using the WST colorimetric assay. A total of 10^5^ cells per well were seeded in 6-well plates before treatment. After 24 h, cells were treated with SH003 (100, 300, and 500 µg/mL), DTX (1, 10, 100, and 1000 nM), or its combination for 24, 48, 72 h. The control medium contained 0.15% ethanol and 0.1% DMSO to offset the effect of each vehicle. No cytotoxic effects were exerted by 0.15% ethanol and 0.1% DMSO on cell growth (data not shown). At the end of the treatment, cells were incubated with WST for 1 h and the absorbance was subsequently measured using an ELISA reader (Molecular Devices, Palo Alto, CA, USA) at 450 nm. 

### 4.4. Colonogenic Assay

H460 cells were seeded at a density of 2 × 104 cells per well in 6-well pates after 24 h treatment with 300 µg/mL SH003, 1 nM DTX, or their combination. After culturing the cells for 5 days, cell colonies were fixed and stained with 0.5% crystal violet solution containing glutaraldehyde for 2 h. After washing off the staining solution, extraction solution used to calculate cell viability [61]. 

### 4.5. Synergism Analysis

Synergistic effects of SH003 and DTX were analyzed using CompuSyn software (Version 3.0.1, CompuSyn Inc., Paramus, NJ, USA). The combination index (CI) was calculated using the following equation [62]:

CIx = (D)1/(Dx)1 + (D)2/(Dx)2(1)

(Dx)1: SH003 dose alone that inhibits x%
(Dx)2: DTX dose alone that inhibits x%
(D)1: The portion of SH003 in combination with SH003 and DTX also inhibits x%
(D)2: The portion of DTX in combination with SH003 and DTX also inhibits x%
“CI < 1”, “CI = 1” and “CI > 1”: mean synergistic, additive, and antagonistic effect, respectively.

### 4.6. Apoptosis Analysis

Apoptosis was analyzed by the double-staining method using FITC-conjugated annexin V apoptosis detection kit and 7-amino-actinomycin D (7-AAD) purchased from BD Pharmingen^TM^ (BD Biosciences, San Jose, CA, USA) and Sigma-Aldrich, respectively. A total of 10^5^ cells/well were cultured in a 6-well plate. Following a 24-h incubation at 37 °C, cells were treated with the considered concentration based on CI value and incubated for 48 h at 37 °C. Cells were collected and resuspended in 1× annexin binding buffer at a concentration of 1 × 10^5^ cells/500 µL, followed by staining with annexin V-FITC or 7-AAD (BD 556570, BD Biosciences). Stained cells were analyzed using FACSCalibur (342973, BD FACSCalibur™, San Jose, CA, USA), and apoptotic cells were analyzed using Cell Quest Pro version 5.2 (BD Biosciences).

### 4.7. Western Blot Analysis

Whole cells were scraped and cell lysates were prepared in lysis buffer (R2002, Biosesang, Seongnam, Korea) containing protease inhibitors. Whole extracts were centrifuged (16,600× *g*, 20 min) and supernatants were obtained. Total protein concentration was estimated using Bradford protein assay (Bio-Rad, Hercules, CA, USA). Equal quantities of proteins were resolved on sodium dodecyl sulfate-polyacrylamide gel and transferred to nitrocellulose membranes in a cold room. After blocking with phosphate-buffered saline-Tween (PBS-T), containing 5% bovine serum albumin (BSA), membranes were incubated at 4 °C overnight with the following primary antibodies: anti-cleaved caspase-3 (#9661; 1:1000), anti-cleaved caspase-7 (#9491; 1:1000), anti-cleaved poly (ADP-ribose) polymerase (PARP; #9541; 1:1000), anti-glyceraldehyde-3-phosphate dehydrogenase (#5174; 1:5000), anti-phospho-EGFR (Tyr 1068), anti-phospho-EGFR (Tyr 1173) (#2236; 1:1000), anti-EGFR (#2232; 1:1000), anti-phospho-STAT3 (#9145; 1:1000), and anti-STAT3 (#4904; 1:1000) purchased from Cell Signaling (Danvers, MA, USA). Membranes were incubated with HRP-conjugated secondary antibodies at 20 °C for 1 h. Secondary antibodies for mouse and rabbit were purchased from SeraCare Life Sciences (Milford, MA, USA).

### 4.8. Immunofluorescence (IF)

Cells were plated on sterile coverslips (3 × 10^4^ cells per coverslip) in 12-well plates. Following treatment with SH003 or DTX for 15 min, cells were fixed with 4% formaldehyde at 20 °C for 10 min. Following fixation, cell membranes were permeabilized with 0.5% Triton X-100 at 20 °C for 7 min. Thereafter, cells were washed with 1× PBS, and the cover slip with a monolayer of cells was blocked with 10% FBS containing 1% BSA and 0.1% Tween-20 in PBS for 2 h, until primary antibody incubation. Rabbit anti-human p-STAT3 antibody diluted at 1:150 in blocking buffer without FBS was added to cells and incubated overnight at 4 °C. Following washing, the cover slip was incubated with AlexaFluor 488 goat anti-rabbit secondary antibody (1:100 dilution) for 1 h 30 min. Slides were washed, counterstained with 4′,6-diamidino-2-phenylindole (1 μg/mL) and mounted with mounting medium. Stained cells were observed under an Olympus FV10i self-contained confocal laser system (Olympus Corporation, Tokyo, Japan).

### 4.9. In Vivo Experiments

All process of animal experiments including maintenance and euthanasia were approved by the Kyung-Hee University Institutional Animal Care and Use Committee (KHU-IACUC-KHSASP-21-211). Five-week-old male Balb/c nude mice were purchased from NARA Biotech (Seoul, Korea) and adapted to a 12 h light/dark cycle at 22–23 °C with 40–60% humidity. The group was divided into four groups (control n = 3, treatment of SH003 n = 4, treatment of DTX n = 4, combination treatment n = 5). Mice were subcutaneously injected with 1 × 10^7^ H460 cells in 100 µL PBS in the right flank. Then, 200 µL SH003 (557.569 mg/kg) was orally administered three times per week and 50 µL DTX (15.277 mg/kg) was intravenously injected through the tail vein once a week for three weeks. Saline and DMSO were used as vehicles. As human-equivalent doses of SH003 and DTX are 4800 mg/kg and 75 mg/m^2^, respectively, the doses were converted to those suitable for animals using the human-equivalent dose formula [63]. The exponent for body surface area (0.75) was determined based on Kleiber’s law [64]. Body weights were measured once every three days while tumor volumes were determined daily using the following formula: tumor volume = length × width^2^ × 0.5. On the 21st day, mice were sacrificed using carbon dioxide, followed by cervical dislocation. Tumor tissues were isolated after euthanasia and photographed.

### 4.10. Histological Analyses

Harvested tumors were fixed with 4% paraformaldehyde overnight, embedded in paraffin blocks, and sectioned at 7 µm. For histological analysis, sectioned tissues were stained with hematoxylin and eosin (H&E) under identical conditions following deparaffinization and rehydration. For immunohistochemical staining, antigen retrieval was performed in 0.01 M sodium citrate buffer (pH 6.0) in a water bath (100 °C) for 10 min, and blocking with normal serum was performed following endogenous peroxidase blocking (Vectastain ABC Kit, Vector Laboratories, United Kingdom). Staining for Ki-67 (1:100; ab16667) and CD31 (1:100; ab28364) purchased from Abcam (Cambridge, MA, USA), cleaved caspase-3 (1:50; #9661), p-EGFR (Y1068; 1:200; #3777), and p-STAT3 (Y705; 1:100; #9145) purchased from Cell Signaling was performed by overnight incubation at 4 °C followed by staining with the secondary antibody for 1 h. Expression of each molecule was analyzed using 3, 3′-diaminobenzidine (DAB) substrate hydrogen peroxide and counterstaining with hematoxylin under identical conditions for each section. Analysis of histological images was processed under a microscope (Carl Zeiss, Jena, Germany) at a magnification of 40×.

### 4.11. Statistical Analysis

Data are represented as the mean ± SEM or SD from at least three experiments with three replicates per experiment. Statistical differences in means between groups were analyzed by one-way ANOVA followed by post-hoc Tukey’s test using Prism v5.03 (GraphPad Prism, San Diego, CA, USA). Results with *p* < 0.05 were considered statistically significant.

## 5. Conclusions

This present study demonstrated that SH003 with DTX is a good combinatorial treatment for lung cancer therapy. Moreover, it may be suggested that SH003 is an effective herbal medical regimen, at least, for EGFR wild-type lung cancer cell lines. Our results provide a strong foundation for clinical trials of SH003 and docetaxel combination therapy for lung cancer patients in whom currently available drugs do not yield favorable outcomes.

## Figures and Tables

**Table 1:** Combination index values of SH003 and docetaxel combinations treatments in H460 cells.

SH003 (μg/mL)	Docetaxel (nM)	Effect	CI Value
100	1	0.577	4.54621
300	1	0.44	0.69068
500	1	0.372	0.77952
100	10	0.516	7.92777
300	10	0.434	1.3307
500	10	0.437	1.77591
100	100	0.485	32.6988
300	100	0.413	4.79899
500	100	0.388	2.87891
100	1000	0.553	2165.1
300	1000	0.5	493.736
500	1000	0.413	43.6589

**Table 2:** Information of human equivalent dose. Animal equivalent dose was calculated using the following equation: human equivalent dose (mg/kg) = animal dose (mg/kg) × (weight_animal_ [kg]/weight_human_ [kg])^(1−0.75)^. The exponent for body surface 0.75 was considered based on the Kleiber’s law.

Docetaxel	Weight	Drug	Dosage	etc.
Human	65 kg	2.023 mg/kg	131.52 mg (75 mg/m^2^)	Exp: 0.75
Mouse	20 g	15.277 mg/kg	0.306 mg	
SH(N7)	Weight	Drug	Dosage	etc.
Human	65 kg	73.946 mg/kg	4800 mg	Exp: 0.75
Mouse	20 g	557.569 mg/kg	11.151 mg	

## Data Availability

The datasets used and/or analyzed during the current study are available from the corresponding author upon reasonable request.
